# Long noncoding RNA ENST00000413528 sponges microRNA‐593‐5p to modulate human glioma growth via polo‐like kinase 1

**DOI:** 10.1111/cns.13121

**Published:** 2019-03-28

**Authors:** Ren Zhang, Ruo‐Lun Wei, Wei Du, Li‐Wei Zhang, Tao Du, Ya‐Dong Geng, Xin‐ting Wei

**Affiliations:** ^1^ Department of Neurosurgery The First Affiliated Hospital of Zhengzhou University Zhengzhou China

**Keywords:** ENST00000413528, glioma, long noncoding RNA, miR‐593, polo‐like kinase 1

## Abstract

**Aims:**

In this study, we examined the expression of lncRNA ENST00000413528 in glioma and determined its role in glioma development.

**Methods:**

LncRNA ENST00000413528 was detected in glioma tissues by lncRNA microarray. Then, we performed real‐time PCR, CCK‐8, colony formation assay, flow cytometry, caspase‐3/7 assay and animal experiment to detect the function of ENST00000413528 in glioma after ENST00000413528 knockdown. Subsequent bioinformatics analysis, luciferase reporter assays and RNA immunoprecipitation (RIP) assay western blotting indicated possible downstream regulatory molecules. The expression of PLK1 in glioma tissues was also examined by immunohistochemistry staining.

**Results:**

Expression of ENST00000413528 was significantly increased in glioma tissues and LN229 and U251 cells. PLK1 protein could not be detected in peritumoral brain edema (PTBE) tissues; however, it showed an increasing number of positively cytoplasmic stained from WHO‐Grade II to Grade III gliomas. Knockdown of ENST00000413528 in glioma cells inhibited cell proliferation and colony formation abilities, induced the G0/G1 arrest of the cell cycle, and promoted apoptosis. The dual reporter assay and RNA immunoprecipitation assay verified the interaction between ENST00000413528 and miR‐593. We also demonstrated that polo‐like kinase 1 (PLK1) was regulated by miR‐593; PLK1 messenger RNA lacking 3’UTR partially reversed the effects caused by ENST00000413528 knockdown or miR‐593 upregulation.

**Conclusion:**

lncRNA ENST00000413528 is closely related to the development of glioma via the miR‐593‐5p/PLK1 pathway.

## 1 INTRODUCTION

Glioma is the most common primary brain tumor and often leads to fatal patient outcomes due to its highly invasive growth pattern and frequent resistance to therapies.^1^, ^2^ In recent years, researchers have increasingly focused on the molecular regulatory networks involved in the development of gliomas and on possible new therapeutic targets. The discovery of mutations in specific genes, such as isocitrate dehydrogenase(IDH) gene mutations,^3^ BRAF mutations,^4^, ^5^ and histone mutations,^6^ has changed our understanding of the pathogenesis of many types of gliomas and subsequently driven the biomarker‐related classification of gliomas.^7^ Notably, the current role of this classification system is not just as a supplement to diagnosis; instead, it goes beyond histological diagnosis.

Human genome sequence data show that most of the transcripts are noncoding ribonucleic acids (RNAs) and that only 2% of the transcripts can encode proteins.^8^ Extensive research has been conducted on microRNAs (miRNAs), confirming their role in the regulation of genes and in the function of cell biology in many cancers.^9^ Recent studies have also shown that long noncoding RNAs (lncRNAs) play an important role in normal development and numerous diseases, including many kinds of cancers.^10^, ^11^ Investigations have also been conducted on lncRNAs and their relevant pathways in glioma. P73 antisense RNA 1T (TP73‐AS1) was upregulated in brain glioma tissues and cell lines and was associated with a poorer prognosis in patients with glioma. Additionally, they determined that TP73‐AS1 may also act as a competing endogenous RNA (ceRNA) to promote HMGB1 expression by sponging miR‐142 to promote the brain glioma growth and invasion.^19^ CeRNA is a known mechanism of lncRNA; in this mechanism, lncRNA competes for posttranscriptional control by sponging certain relevant miRNAs.^20^ Previous surveys have confirmed some lncRNA‐miRNA interaction pathways, 21, 22 which provide some theoretical basis for our current research.

Polo‐like kinase 1 (PLK1) is a serine/threonine protein kinase that is known to be a key regulator of mitosis, with critical roles in cell cycle‐associated processes.^25^, ^26^ PLK1 is involved in the regulation of the cell cycle and the growth of various tumors, including breast cancer, renal cell cancer, and prostate cancer.^18^, ^27^, ^28^ Two studies found some lncRNAs, such as Hox transcript antisense RNA (HOTAIR) and lnc‐RI, exerted their regulatory effects through PLK1^31^, ^32^; however, such lncRNA‐PLK1 associated approach has not yet been observed in glioma cells.

In this study, we performed a lncRNA microarray in glioma tissues and found lncRNA ENST00000413528 was overexpressed. After the qRT‐PCR verification in glioma tissues and biological informational analysis, we found a possible ceRNA pathway of lncRNA ENST00000413528, which shared a complementary area with miR‐593‐5p, and associated with the target gene PLK1. We, thus, hypothesized that lncRNA ENST00000413528 may sponge miR‐593‐5p to regulate PLK1 to exert its effects on glioma cells and designed a series of experiments to verify our hypothesis.

## 2 MATERIALS AND METHODS

### 2.1 Ethics statement

The human studies included in this research were conformed to Declaration of Helsinki; both the human and animal studies were approved by the medical research ethics committee of the First Affiliated Hospital of Zhengzhou University in Zhengzhou, China. All patients with glioma signed an informed consent prior to participation.

### 2.2 Cells and tissues

The glioma cell lines U251, SNB19, U373, SHG44, and LN229 and normal human astrocytes (NHAs) were obtained from the American Type Culture Collection (Manassas, VA, USA). Cells were cultured in a humidified atmosphere with 5% CO_2_ at 37°C in an incubator (Thermo Fisher Scientific, Waltham, MA, USA). High‐glucose Dulbecco's Modified Eagle's medium (DMEM; Merck KGaA, Darmstadt, Germany) with 10% fetal bovine serum (FBS; Gibco, Waltham, MA, USA) and 1% penicillin‐streptomycin (Solarbio, Beijing, China) were used for cell culture. Trypsin (0.25%) (Solarbio, Beijing, China) was used to dissociate the cells. Sixteen brain glioma and paired peritumoral brain edema (PTBE) tissues were collected following tumor surgical resection performed at the First Affiliated Hospital of Zhengzhou University. Collected tissues were immediately placed into liquid nitrogen. The primary antibodies for western blotting or immunohistochemistry against glyceraldehyde 3‐phosphate dehydrogenase (GAPDH) (ab8245) and PLK1 (ab17056), as well as secondary antibody (ab6789), were purchased from Abcam (Cambridge, UK).

### 2.3 LncRNA microarray of glioma tissues

Total RNA of 3 paired human brain glioma and PTBE tissues was extracted and purified using the mirVana™ miRNA Isolation Kit (Ambion, Austin, TX, USA) according to the manufacturer's instructions. The total RNA was qualified by the NanoDrop™ ND‐2000 (Thermo Fisher Scientific, Waltham, MA, USA) and the Agilent Bioanalyzer 2100 (Agilent Technologies, Santa Clara, CA, USA) for subsequent chip experimentation. Shanghai Biotechnology Cooperation (Shanghai, China) carried out the following assays using the Agilent Human lncRNA V6 chip (Agilent Technologies, Santa Clara, CA, USA) per the manufacturer's instructions. Slides were scanned using the Agilent Microarray Scanner (Agilent Technologies, Santa Clara, CA, USA), while data were extracted with the Feature Extraction software version 10.7 (Agilent Technologies, Santa Clara, CA, USA).

### 2.4 RNA extraction and quantitative real‐time polymerase chain reaction

Total RNA in cells and tissues was extracted using TRIzol (Invitrogen, Carlsbad, CA, USA) and qualified by use of the NanoDrop™ ND‐2000 (Thermo Fisher Scientific, Waltham, MA, USA). After the total RNA reverse transcription to cDNA by use of the First Strand cDNA Synthesis Kit (Thermo Scientific, Waltham, MA, USA), qRT‐PCR was carried out to determine the relative expression levels of lncRNA ENST00000413528, lnc‐NUDT4‐4:1, NONHSAT207178, NONHSAT174301, and miR‐593‐5p using the 7500 Fast PCR instrument (Applied Biosystems, Foster City, CA, USA) with the reagent of Maxima SYBR Green qPCR Master Mix (2×) (Thermo Fisher Scientific, Waltham, MA, USA). GAPDH and U6 were used as the internal control for lncRNA and miRNAs, respectively. Relative expression levels of RNAs were calculated as 2^−ΔΔCT^.^33^ Primer sequences were synthesized by Shanghai Biological Technology Cooperation (Shanghai, China).

### 2.5 Protein extraction and western blotting

Tissues and cells were lysed by radioimmunoprecipitation assay (RIPA) lysis buffer (Solarbio, Beijing, China) to obtain the total protein, while a bicinchoninic acid (BCA) protein assay kit (Beyotime, Shanghai, China) was used to draw the standard curve and calculate the protein concentration of each sample. After the preparation of the 10% sodium dodecyl sulfate‐polyacrylamide (Solarbio, Beijing, China) separation gel and 5% concentration gel, a protein sample (30 μg), which was adjusted to the same concentration by the loading buffer (Solarbio, Beijing, China), was added to the electrophoretic sample hole. The samples were then subjected to vertical electrophoresis with a 120 V constant voltage for between 1 and 2 hours, after which a polyvinylidene difluoride filter (PVDF) membrane (Thermo Fisher Scientific, Waltham, MA, USA) was used to transfer the protein band by use of a “sandwich” structure with a 200 mA constant current in electrophoresis. The PVDF membrane was subsequently blocked at room temperature by 5% skim milk powder for 2 hours. The membrane was washed with Tris‐buffered saline containing Tween 20 (TBST; Solarbio, Beijing, China) four times before being incubated overnight with the primary antibody for GAPDH or PLK1 (1:1000 dilutions) at 4°C. After washing the membrane again with TBST four times after the primary antibody incubation, the membrane was incubated with a secondary goat antimouse antibody (1:10000 dilutions) for 1 hour at room temperature. The membrane was washed again (following the same procedure as before). The ECL Western Blotting Substrate (Thermo Scientific, Waltham, MA, USA) was then dropped on the membrane, and the protein immunoreactivity was determined by FluorChem E (ProteinSimple, San Jose, CA, USA).

### 2.6 Cell transfection

Short hairpin RNA (ShRNA) lentivirus fluid that interfered with lncRNA ENST00000413528 expression (si‐Lnc) or its negative control (si‐NC) was constructed and purified by Hanheng Biotechnology Corporation (Shanghai, China), and puromycin was subsequently used to select stably infected cells. SiRNA that interfered with PLK1 expression, miR‐593 mimics, or negative control of miR‐593 (Genepharma, Shanghai, China) was transfected into cells using Lipofectamine 2000 (Invitrogen, Carlsbad, CA, USA) when the cell density was 70% to 90%. The expression vector of PLK1 lacking 3’UTR (pcDNA3.1‐PLK1; Hanheng Biotechnology Corp., Shanghai, China) was also transfected into cells by use of Lipofectamine 2000 (Invitrogen, Carlsbad, CA, USA) according to the manufacturer's instructions.

### 2.7 Cell Counting Kit‐8 assay

Cells were dissociated and seeded in 96‐well plates at the density of 2 × 10^4^/well, and siRNA or si‐NC was transfected into cells when the cell confluence was approximately 70%. After 24, 48, or 72 hours of incubation, the medium was replaced by 10 μL of the Cell Counting Kit‐8 (CCK‐8; Dojindo, Kumamoto, Japan) solution and 90 μL of DMEM with 10% FBS. The absorbance value of 450 nm was detected under a microplate reader (Bio‐RAD, Hercules, CA, USA) 2 hours later.

### 2.8 Cell colony formation assay

Cells with different treatments were seeded in the 3.5 cm cell culture dish (Corning, Corning, NY, USA) at the density of 150/dish (with 2 mL DMEM with 10% FBS). Then, 200 μL of DMEM with 10% FBS was added into the dish every 2 days to supplement the medium that volatilized into the incubator. Cells were incubated for 10 to 14 days until the colonies were visible to the naked eye on the bottom of the culture dish. After removing the medium, cell colonies were fixed with methanol for 30 minutes and then stained with 0.1% crystal violet (Solarbio, Beijing, China). The number of colonies stained in the dish for analysis was then counted, and the experiment was repeated three times.

### 2.9 Cell apoptosis assays

The number of apoptotic cells of each group was detected via the Annexin V‐FITC/PI Apoptosis Detection Kit (KenGen BioTECH, Jiangsu, China). Cells (1‐5 × 10^5^) were dissociated with 0.25% trypsin without ethylenediaminetetraacetic acid (EDTA; Solarbio, Beijing, China), washed with PBS, and resuspended with 500 μL of the binding buffer. Next, 5 μL of annexin V‐FITC (Fluorescein isothiocyanate) and PI (Propidium iodide) was added into the binding buffer and fully mixed. After incubating, light was avoided at room temperature for 5 to 15 minutes; the cells were then detected and classified as living cells, dead cells, early apoptotic cells, and late apoptotic cells using flow cytometry (BD, San Diego, CA, USA), with channel FL1 used for annexin V‐FITC and channel FL3 used for PI detection, respectively.

The Apo‐ONE^®^ Homogeneous Caspase‐3/7 Assay Kit (Promega, Madison, WI, USA) was used to detect caspase 3/7 activity. Cells were seeded and incubated for 24 hours after treatment in 96‐well plates and then Apo‐ONE^®^ Caspase 3/7 Reagent was added (100 μL), mixed with the medium, and incubated for 3 hours with a plate sealer cover. The fluorescence (RFU), at an excitation wavelength of 499 nm and an emission wavelength of 521 nm of each well, was then measured by a spectrofluorometer (Thermo Fisher Scientific, Waltham, MA, USA). Caspase 3/7 activity = RFU_(experimental group)_ / RFU_(NC group)_ × 100%.

### 2.10 Cell cycle detection

Cell cycle status of each group was detected by flow cytometry using a cell cycle detection kit (KenGen BioTECH, Jiangsu, China). Cells (1 × 10^6^) were dissociated with 0.25% trypsin without EDTA, washed twice with PBS, and fixed with 500 μL of 70% precooled ethanol for 2 hours. After fixation, the cells were washed with PBS once; 500 μL of the working solution (RNaseA: PI = 1:9) was then added to resuspend cells. After incubating cells in the dark for 30 to 60 minutes at room temperature, the red fluorescent signal at the excitation wavelength of 488 nm was detected. The analysis was performed and figures were created using Modfit software.

### 2.11 Dual luciferase reporter assay

In order to verify the interaction between LncRNA ENST00000413528 and miR‐593, miR‐593, and PLK1, we conducted the dual luciferase reporter assay. The wild and mutant target fragments of LncRNA ENST00000413528 and PLK1 were obtained by PCR (2× Taq PCR Mix; Tiangen, Beijing, China) and connected with T vector (Tiangen, Beijing, China). After transformation and identification, the correct recombinant plasmid was selected for double enzyme cutting (TaKaRa, Dalian, China). The target fragments after digestion were then connected with pmirGLO plasmid (Promega, Madison, WI, USA), which was digested by the same enzymes. Then, following transformation and identification, the correct recombinant plasmid (ie pmirGLO‐Wt‐Lnc, pmirGLO‐Mt‐Lnc, pmirGLO‐Wt‐PLK1, or pmirGLO‐Mt‐PLK1) was cotransfected into either cells with miR‐593 mimics or miR‐NC using lipofectamine 2000. Cells were lysed for 24 hours after transfection and transferred to 96‐well plates. The luciferase activity of the renilla and firefly of each well was detected using a luciferase assay kit (Promega, Madison, WI, USA) and Centro XS^3^ LB 960 Microplate Luminometer (Berthold, Bad Wildbad, Germany). The ratio of the luciferase activity of firefly and renilla was calculated and analyzed.

### 2.12 Animal experiment

A xenograft mouse model was constructed to observe the effects of LncRNA ENST00000413528 knockdown on tumor proliferation in U251 cells in vivo. The female specific pathogen‐free (SPF) grade nude mice (n = 16, BALB/c, 4‐6 weeks old, 14‐17 g) from the Beijing Vital River Laboratory Animal Technology Center (Beijing, China) were given laboratory chow and water in a SPF‐grade animal room. si‐Lnc or si‐NC infected U251 cells (5 × 10^6^) were injected into the left armpit of the mice. The mice were observed weekly to record their weight and tumor volume (volume = 1/2 × length × width^2^) and sacrificed 4 weeks after implantation.

### 2.13 RNA immunoprecipitation assay

RNA immunoprecipitation (RIP) assay was performed using the Magna RNA‐Binding Protein Immunoprecipitation Kit (Merck KGaA, Darmstadt, Germany) and the Ago2 antibody (ab32381; Abcam, Cambridge, UK) according to the manufacturer's protocols. After the antibody was recovered by protein beads, qRT‐PCR was performed to detect the relative expression level of lncRNA ENST00000413528 and miR‐593 in the precipitates.^34^

### 2.14 Immunohistochemistry (IHC) and hematoxylin and eosin (H&E) staining

Samples were dehydrated in gradient alcohol, fixed in neutral‐buffered formalin and embedded in paraffin. They were prepared into 4‐6‐μm‐thick tissue sections and then dewaxed. For H&E staining, hematoxylin and eosin were stained, rinsed by water, then dehydrated, and sealed by neutral gum as the manufacturer's instructions (Solarbio, Beijing, China). For IHC staining, antigen retrieval was performed by pressure cooking at 100°C in 0.01M Sodium Citrate buffer for 20 minutes. Slides were then treated with 3% H_2_O_2_ in methanol. After blocking, the samples were incubated with the first PLK1 antibody diluted 1:500 at 37°C for 1 hours. The rest procedure of immunohistochemistry was performed as the manufacturer's instructions (Absin, Shanghai, China), and the final results were determined by two pathologists independently.

### 2.15 Statistical analysis

All cell experiments were repeated three times. SPSS 21 software (IBM Corp., Armonk, NY, USA) was used to perform the data analysis. Data were expressed in the format of the mean ± standard deviation (SD). A Student's *t* test, one‐way analysis of variance, Pearson's analysis was carried out to analyze the data. *P* < 0.05 was considered statistically significant.

## 3 RESULTS

### 3.1 Detection and verification of lncRNA microarray in glioma tissues

**Figure 1:**
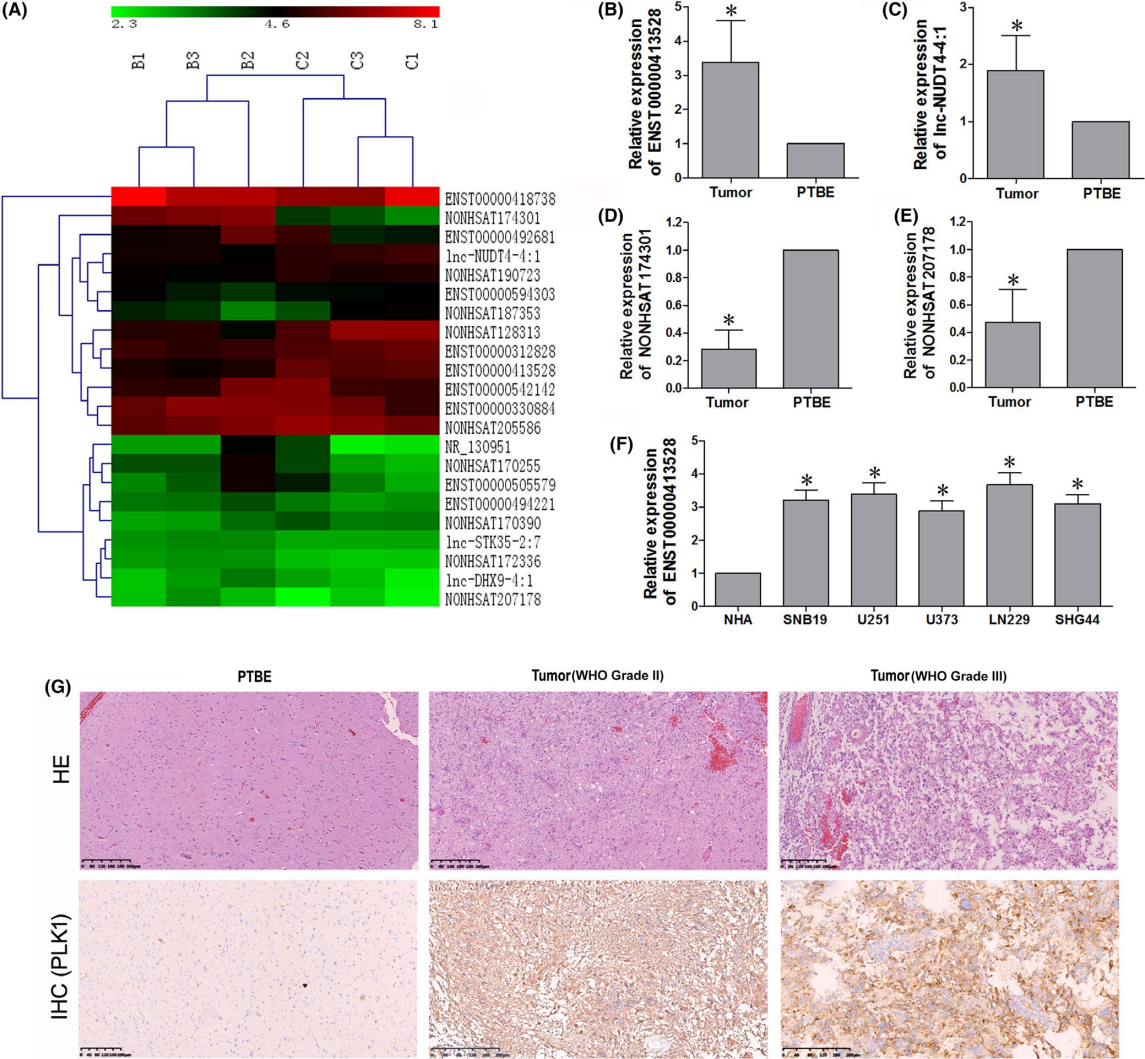
A, Differential expressed lncRNAs in glioma tissues detected by the Agilent Human lncRNA V6 chip (Agilent Technologies, Santa Clara, CA, USA). B refers to the brain glioma tissue; C refers to the control tissue (peritumoral brain edema tissue). B, C, D, E, Verification of four differential expressed lncRNAs in 16 pairs of glioma and paired peritumoral brain edema (PTBE) tissues using qRT‐PCR with the 2^−ΔΔCT^ method. F, Relative expression level of lncRNA ENST00000413528 in different glioma cells as detected by qRT‐PCR. Each assay was performed three times. G, HE and PLK1 IHC staining in PTBE and grade II, III glioma tissues. The positive staining was majorly localized in the cytoplasm of glioma tissues, grade III glioma expressed a higher level of PLK1. IHC staining showed no detectable PLK1 expression in PTBE tissues

The microarray of three human brain glioma tissues and the paired PTBE tissues showed 22 differential expressed lncRNAs, including 13 downregulated and nine upregulated ones (Figure 1A, Table 1). LncRNA ENST00000413528 was found to demonstrate a 3.58‐fold change upregulation in the glioma specimens. To verify the microarray results, we performed the qRT‐PCR of two upregulated and two downregulated lncRNAs in 16 paired human glioma tissues and their corresponding PTBE tissues (Figure 1B,C,D,E). Our results showed that the relative expression of ENST00000413528 or lnc‐NUDT4‐4:1 was higher in tumor tissues than that in PTBE tissues (*P* < 0.05), while NONHSAT207178 and NONHSAT174301 were downregulated in tumor tissues (*P* < 0.05), all of which were consistent with the microarray results. Thus, we confirmed that the data of the microarray were statistically credible. In addition, the morphological changes in glioma tissues were displayed by H&E staining in Figure 1G. PLK1, the targeted gene in our hypothesis, was also detected by IHC. PLK‐1 protein could be mainly cytoplasmic stained in glioma tissues while hardly stained in PTBE tissues. The PLK1 expression showed a significantly and gradually increased tendency from PTBE, low‐grade glioma to high‐grade glioma tissues. We then detected the relative expression level of ENST00000413528 in different glioma cell lines for further study (Figure 1F). Expression of ENST00000413528 was significantly increased in glioma cell lines, especially in LN229 and U251 cells, when compared with NHA cells (*P* < 0.05). Therefore, we selected LN229 and U251 cells to carry out the next experiments.

**Table 1:** Significantly aberrantly expressed lncRNAs determined by microarray

LncRNA	FC	Regulation	Chromosomal location	*P*
lnc‐STK35‐2:7	2.09	Down	chr20	0.0188
NONHSAT172336	2.19	Down	chr151	0.0341
NONHSAT207178	2.21	Down	chr6	0.0310
ENST00000418738	2.22	Down	chr11	0.0203
NONHSAT205586	2.28	Down	chr5	0.0142
ENST00000494221	2.32	Down	chr7	0.0197
ENST00000505579	2.46	Down	chr2	0.0182
lnc‐DHX9‐4:1	2.52	Down	chr1	0.0213
ENST00000330884	2.62	Down	chr22	0.0170
ENST00000492681	2.71	Down	chr3	0.0482
NR_130951	3.13	Down	chr10	0.0041
NONHSAT170255	3.34	Down	chr14	0.0321
NONHSAT174301	6.70	Down	chr16	0.0372
ENST00000542142	2.06	Up	chr12	0.0095
NONHSAT190723	2.20	Up	chr21	0.0008
NONHSAT170390	2.29	Up	chr14	0.0144
ENST00000594303	2.36	Up	chr19	0.0218
NONHSAT187353	2.73	Up	chr2	0.0109
lnc‐NUDT4‐4:1	3.15	Up	chr12	0.0108
ENST00000312828	3.27	Up	chr18	0.0282
ENST00000413528	3.58	Up	chrX	0.0010
NONHSAT128313	5.83	Up	chr8	0.0317

### 3.2 Knockdown of ENST00000413528 inhibited cell proliferation and induced apoptosis and cell cycle arrest in glioma cells

**Figure 2:**
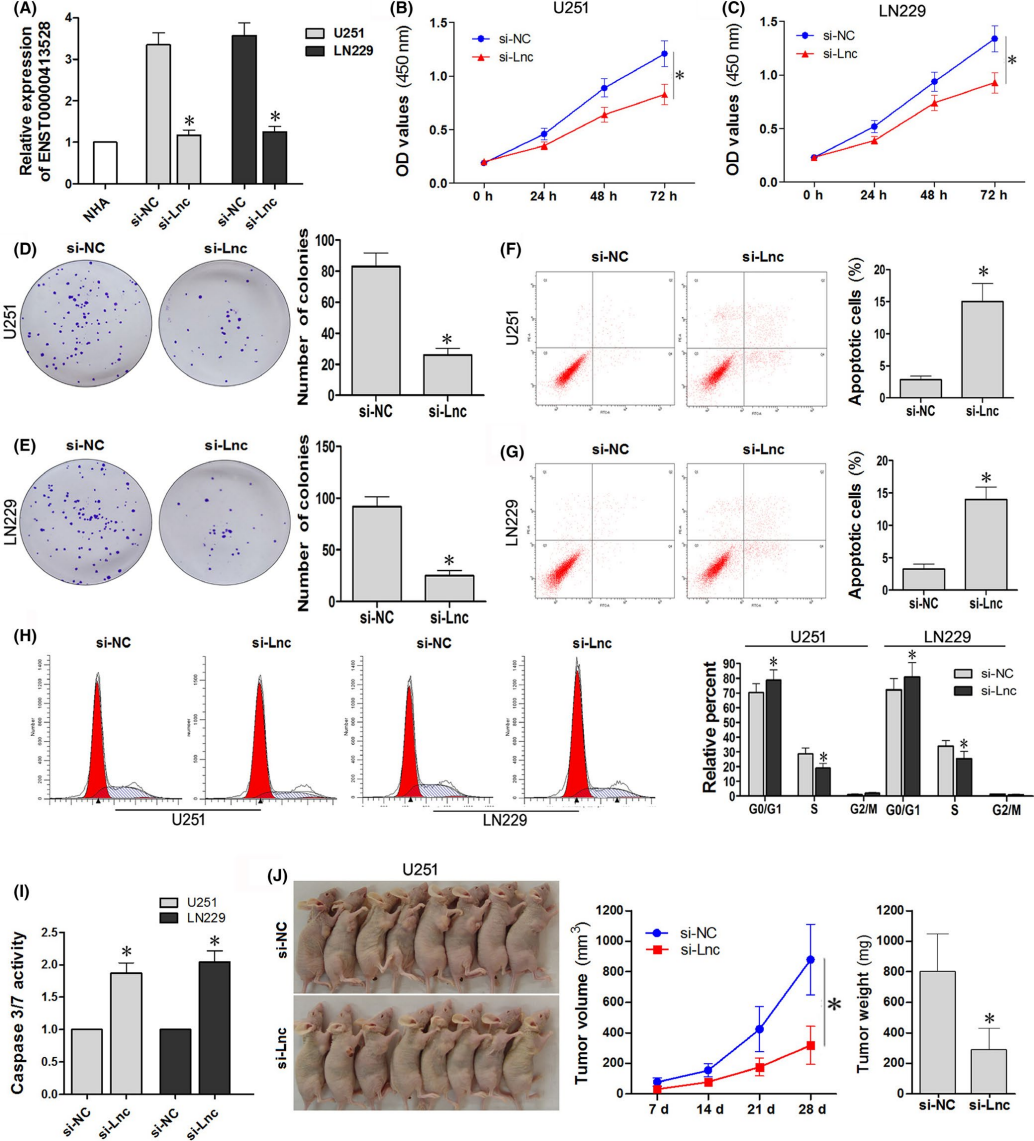
Effects of ENST00000413528 on proliferation, colony formation, apoptosis, and cell cycle in glioma cells. A, Expression level verification of ENST00000413528 in glioma cells after lentivirus fluid containing shRNA‐Lnc or shRNA‐NC infection and puromycin selection. B,C, The CCK‐8 assay was performed at 0, 24, 48, and 72 h in U251 and LN229 cells, respectively, and the optical density (OD) value detected by microplate reader of each group was recorded. D,E, Number of cell colonies of the two groups in the U251 and LN229 cells. F, G, Number of apoptosis cells detected by the flow cytometry of the two groups in the U251 and LN229 cells. H, Cell cycle status and relative percentage of cells in the two groups in the U251 and LN229 cells. I, Caspase 3/7 activity comparison in the two groups. J, A xenograft mouse model experiment was constructed using U251 cells with the si‐Lnc or si‐NC infection; the tumor volume and mouse weight of each mouse was recorded

LN229 and U251 cells were infected by lentivirus fluid containing si‐Lnc or si‐NC, and puromycin was used to select cells which may stably knockdown ENST00000413528 expression. We evaluated the relative expression of ENST00000413528 in different groups (Figure 2A), which showed an obvious downregulation in cells infected with si‐Lnc lentivirus fluid when compared with si‐NC‐infected cells (*P* < 0.05). CCK‐8 assay showed that the optical density (OD) value of the si‐Lnc cell group was significantly lower than that of the si‐NC cell group (Figure 2B,C; *P* < 0.05), suggesting that the downregulation of ENST00000413528 inhibits the growth of LN229 and U251 cells. The colony formation assay illustrated that the ability of colony formation was weakened in cells with downregulated ENST00000413528, via showing a decrease in the number of cell colonies when compared with the si‐NC group (Figure 2D,E; *P < *0.05). In addition, more apoptotic cells were detected in si‐Lnc groups than in si‐NC groups (Figure 2F,G; *P < *0.05), and a similar difference also occurred in caspase 3/7 activity of LN229 and U251 cells (Figure 2I; *P* < 0.05). Furthermore, we also assessed the cell cycle status in the two groups using flow cytometry and found that cells infected with si‐Lnc had increased cell numbers in the G0/G1 phases, but reduced numbers in the S phase (Figure 2H; *P* < 0.05), suggesting that si‐Lnc‐related cell cycle arrest occurred in the G0/G1 checkpoints for LN229 and U251 cells. Glioma nude mice models constructed by si‐Lnc or si‐NC U251 cells were performed to evaluate the effects of ENST00000413528 on glioma cell growth in vivo. Results showed that the tumor volume and tumor weight of mice in the si‐Lnc group were both significantly decreased when compared with these factors in the si‐NC group (Figure 2J; *P* < 0.05). Therefore, our results suggest that the downregulation of lncRNA ENST00000413528 may impair glioma cell growth*,* promote cell apoptosis in LN229 and U251 cells, and the proliferation inhibitory effect was also demonstrated in our xenograft mouse model constructed using U251 cells.

### 3.3 LncRNA ENST00000413528 regulated miR‐593‐5p by direct targeting

**Figure 3:**
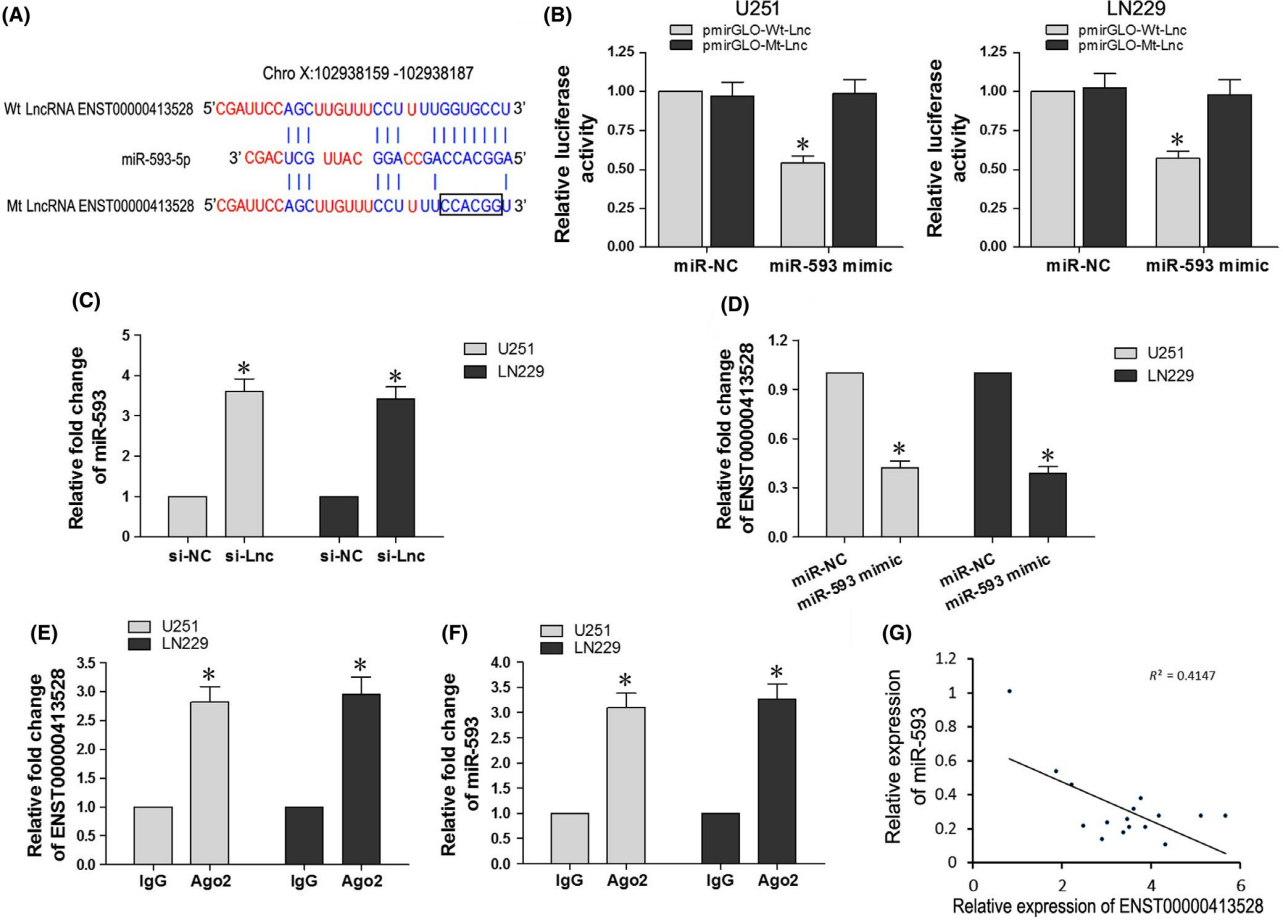
LncRNA ENST00000413528 regulated miR‐593‐5p by direct targeting. A, A complementary pairing area was found between ENST00000413528 and miR‐593, miR‐593, and PLK1. B, pmirGLO‐Wt‐Lnc or pmirGLO‐Mt‐Lnc was cotransfected with miR‐593 mimics or miR‐NC, respectively, and the luciferase activity of each group was detected. C, Relative expression level of miR‐593 when ENST00000413528 was knocked down. D, Relative expression level of ENST00000413528 when miR‐593 was upregulated. E, Immunoprecipitation of Ago2‐associated ENST00000413528 was verified by RT‐qPCR. Immunoglobulin G (IgG) served as the negative control. F, Immunoprecipitation of Ago2‐associated miR‐593 was verified by RT‐qPCR. IgG served as the negative control. G, Correlation analysis between the relative expression levels of ENST00000413528 and miR‐593 in 16 pairs of glioma and paired peritumoral brain edema (PTBE) tissues. Each assay was performed three times

Bioinformatics analysis demonstrated a complementary pairing area between the sequences of ENST00000413528 and miR‐593‐5p (Figure 3A). To investigate whether miR‐593‐5p was the target gene of ENST00000413528, we constructed recombinant plasmid pmirGLO‐Wt‐Lnc and pmirGLO‐Mt‐Lnc, which was then cotransfected with miR‐NC or miR‐593 mimics into U251 and LN299 cells for the performance of the dual luciferase reporter assay (Figure 3B). Our results showed that the luciferase activity of the Wt‐Lnc luciferase reporter vector was notably suppressed in response to the miR‐593 mimic transfection when compared with the NC group (*P* < 0.05), suggesting a direct combination between miR‐593 and the Wt‐Lnc sequence. To clarify the regulation effects between miR‐593 and ENST00000413528, we then detected the relative expression of miR‐593 in si‐Lnc cells or si‐NC cells. Interestingly, our results showed that the downregulation of lncRNA ENST00000413528 significantly increased miR‐593 expression in both U251 and LN299 cells when compared with NC cells (Figure 3C; *P* < 0.05). Similarly, the upregulation of miR‐593 could also lead to a decrease of ENST00000413528 expression (Figure 3D; *P* < 0.05). We performed RIP assay to clarify whether ENST00000413528 and miR‐593 exist in the RNA‐induced silencing complex (RISC); qRT‐PCR was then performed to detect Ago2‐associated RNAs in the precipitates. Ago2‐associated ENST00000413528 had a more than 2.5‐fold enrichment and miR‐593 had a more than 3‐fold enrichment when compared with immunoglobulin G (IgG) (Figure 3E,F; *P* < 0.05). Correlation analysis between the relative expression of miR‐593 and ENST00000413528 in glioma tissues demonstrated a negative correlation with *R* ^2^ at 0.4147 (Figure 3G; *P* < 0.05). Overall, the results of the dual luciferase reporter assay, RIP assay, and correlation analysis suggested that LncRNA ENST00000413528 directly targeted miR‐593‐5p.

### 3.4 PLK1 was a target gene of miR‐593‐5p

**Figure 4:**
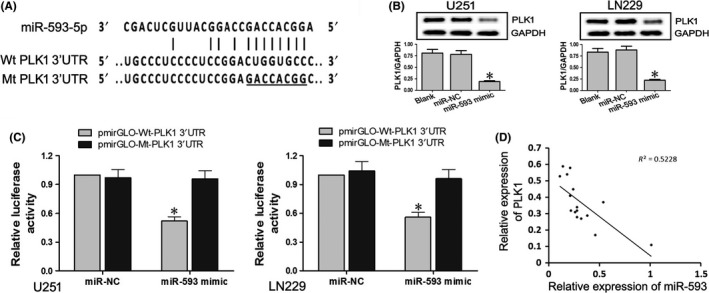
PLK1 was a target gene of miR‐593‐5p. A, A complementary pairing area or mutant area was shown between miR‐593 and 3′UTR PLK1 with the wild type or mutant type, respectively. B, The relative expression level of the PLK1 protein in cells transfected with miR‐593 mimics, miR‐NC, or blank control was detected using western blotting, with GAPDH serving as an internal reference. C, pmirGLO‐Wt‐PLK1 3′UTR or pmirGLO‐Mt‐PLK1 3′UTR was cotransfected with miR‐593 mimics or miR‐NC, respectively, in the U251 and LN229 cells, and the luciferase activity of each group was detected. D, Correlation analysis between the relative expression levels of PLK1 mRNA and miR‐593 in 16 pair of glioma and paired peritumoral brain edema (PTBE) tissues

MiRNAs regulate gene expression by binding to the three prime untranslated regions (UTRs) of their target genes. We performed the bioinformatics analysis and found a complementary pairing area between the sequences of miR‐593‐5p and PLK1 (Figure 4A). To verify whether PLK1 was a target gene of miR‐593‐5p, we performed the dual luciferase reporter assay. Recombinant plasmids pmirGLO‐Wt‐PLK1 3′UTR and pmirGLO‐Mt‐PLK1 3′UTR were cotransfected with miR‐NC or miR‐593 mimics into U251 and LN299 cells (Figure 4C). Our results showed that the luciferase activity of the Wt‐PLK1 luciferase reporter vector was notably suppressed in response to the miR‐593 mimics' transfection when compared with the NC group (*P* < 0.05), indicating a direct combination between miR‐593 and PLK1 3′UTR. Subsequently, the expression of the PLK1 protein in cells transfected with miR‐593, NC control, or blank control was detected by western blotting (Figure 4B). The upregulation of miR‐593 significantly decreased the PLK1 protein expression (*P* < 0.05). Moreover, in glioma tissues, the relative expression of PLK1 protein was negatively correlated with miR‐593 (*R* ^2^ = 0.5228, *P* < 0.05). The results from dual luciferase reporter assay, western blotting, and tissue verification suggested that miR‐593‐5p negatively regulated PLK1 by targeting PLK1 3′UTR. We inferred that lncRNA ENST00000413528 may function as a ceRNA in miR‐593‐5p/PLK1 pathway when combining the results 3.3 and 3.4.

### 3.5 Consistent effects of lncRNA ENST00000413528 downregulation and miR‐593 upregulation on the cell growth, cell cycle, and apoptosis in U251 and LN229 cells

**Figure 5:**
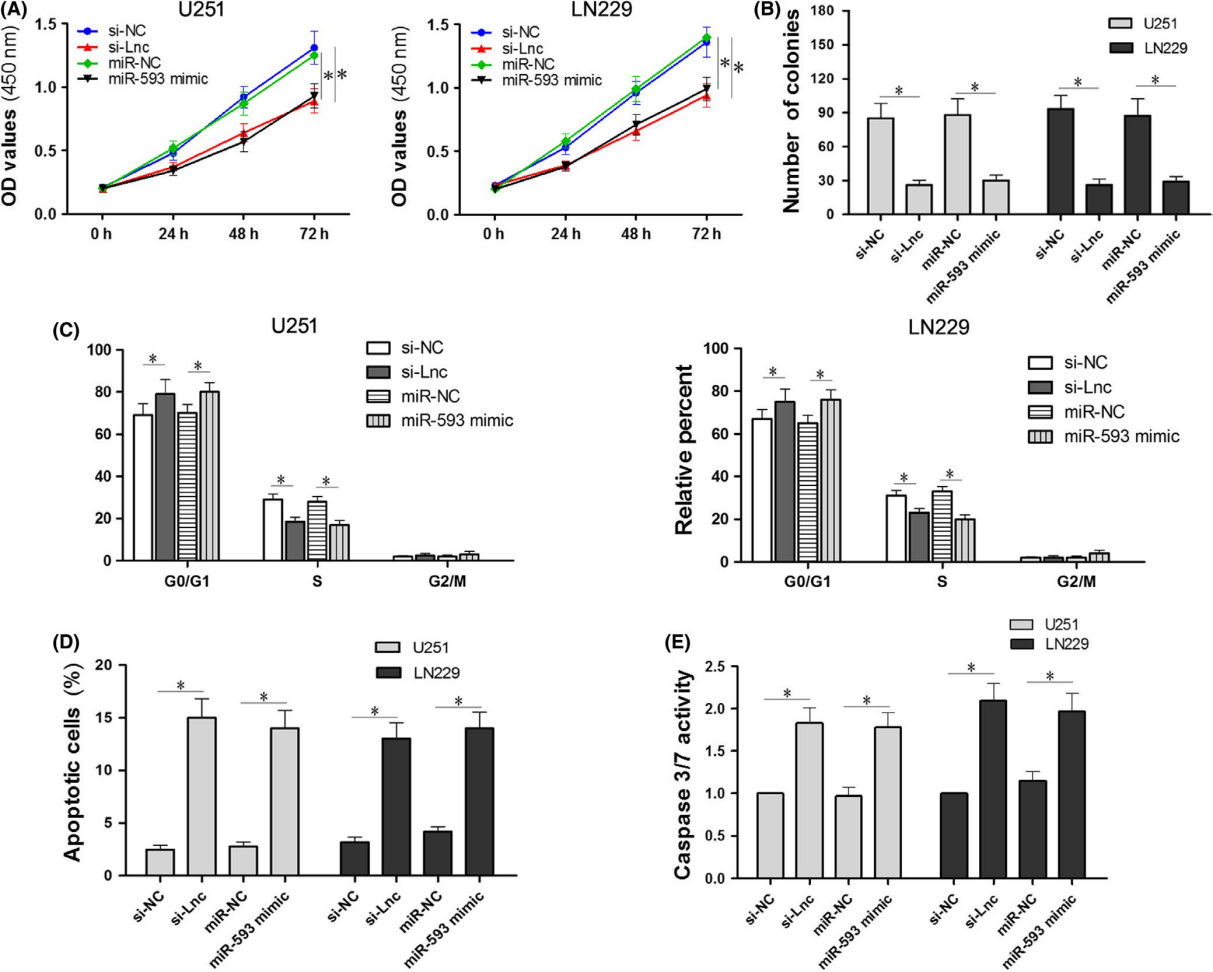
Effects comparison between ENST00000413528 downregulation and miR‐593 upregulation. Cells were divided into four groups with either the si‐Lnc or si‐NC lentivirus infection, miR‐593 mimics, or miR‐NC transfection. A, The CCK‐8 assay was performed at 0, 24, 48, and 72 h after transfection in the U251 and LN229 cells, respectively, and the OD value as detected by the microplate reader of each group was recorded. B, The number of cell colonies in the four groups was compared. C, The relative percentages of the 3‐cell cycle status in the four groups in the U251 and LN229 cells were compared. D, The number of apoptosis cells was detected by flow cytometry of the four groups in the U251 and LN229 cells. E, Caspase 3/7 activity was compared in the four groups

We have demonstrated that the downregulation of ENST00000413528 affected the growth and apoptosis of U251 and LN229 cells and that there was a negative correlation between ENST00000413528 and miR‐593. We then verified a regulation pathway via ENST00000413528/miR‐593/PLK1. Therefore, to further clarify the effects of ENST00000413528 and miR‐593, we designed four cell groups (ie, cells with the ENST00000413528 knockdown or miR‐593 upregulation and their respective negative controls) to carry out the following experiments. The CCK‐8 assay showed similar proliferation inhibitory effects in si‐Lnc and miR‐593 mimics cell groups when compared with the NC groups (Figure 5A; *P* < 0.05). A significant decrease in the number of colonies in U251 and LN229 cells was observed with either downregulating ENST00000413528 or upregulating miR‐593 (Figure 5B; *P* < 0.05). We also found that the cell cycle status was altered by transfecting with si‐Lnc or miR‐593 mimics versus that found in the NC groups, with an inhibition occurring in the S phase and a relative percent increase happening in the G0/G1 phase; furthermore, there was no significant difference in the effects of the ENST00000413528 knockdown and miR‐593 overexpression on cell cycle regulation in U251 and LN229 cells (Figure 5C; *P* < 0.05). For antiapoptosis effects, we evaluated the apoptosis cells (Figure 5D) and caspase 3/7 activity (Figure 5E) in the 4 groups. The number of apoptosis cells declined significantly in both the si‐Lnc and miR‐593 mimic groups (*P* < 0.05). The caspase 3/7 activity increased in the 2 groups but did not result in a significant difference within the two groups (*P* < 0.05).

### 3.6 Reversed effects of PLK1 on ENST00000413528/miR‐593/PLK1 signaling

**Figure 6:**
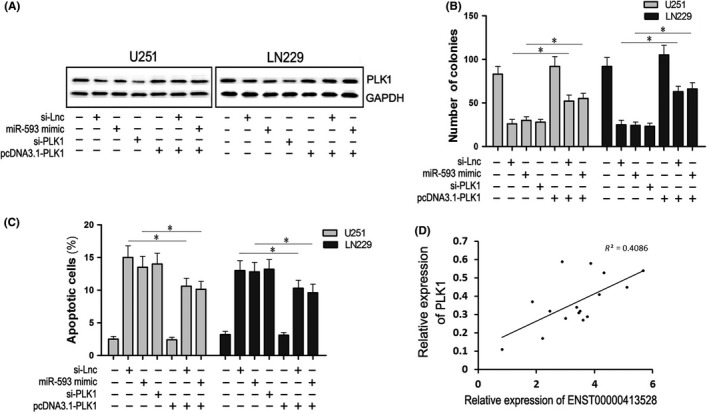
Reversed effects of PLK1 on ENST00000413528/miR‐593/PLK1 signaling. Cells were divided into seven groups: a blank group without any treatment; an si‐Lnc group with an si‐Lnc lentivirus fluid stable infection; an miR‐593 group with miR‐593 mimic transfection; an si‐PLK1 group with si‐PLK1 transfection; a pcDNA3.1‐PLK1 group with pcDNA3.1‐PLK1 (lacking 3′UTR) transfection; an si‐Lnc + pcDNA3.1‐PLK1 group with pcDNA3.1‐PLK1 transfected into si‐Lnc stably infected cells; and an miR‐593 + pcDNA3.1‐PLK1 group with miR‐593 mimics and pcDNA3.1‐PLK1 cotransfection. A, The relative expression levels of the PLK1 protein in the seven groups, with GAPDH serving as an internal reference. B, The number of cell colonies in the seven groups. C, The number of apoptosis cells detected by flow cytometry in the seven groups. D, Correlation analysis between the relative expression levels of PLK1 mRNA and ENST00000413528 in 16 pair of glioma and paired peritumoral brain edema (PTBE) tissues

Our previous data demonstrated that ENST00000413528 might act as a ceRNA to promote PLK1 expression by sponging miR‐593; we performed a restoration experiment for further verification. Recombinant plasmid pcDNA3.1‐PLK1 lacking 3’UTR, which was not able to bind to miR‐593, was constructed and transfected into U251 and LN229 cells alone, cotransfected with miR‐593 mimics, or transfected into si‐Lnc cells. The PLK1 expression level (as detected by western blotting) showed a downregulation effect in the cells of the si‐Lnc, miR‐593 mimics, and si‐PLK1 groups and an upregulation effect in the pcDNA3.1‐PLK1 group (Figure 6A;* P* < 0.05). It is worth noting that, when pcDNA3.1‐PLK1 was transfected into si‐Lnc cells or cotransfected with miR‐593 mimics into U251 and LN229 cells, the knockdown effects of the 2 were partially reversed by pcDNA3.1‐PLK1. The numbers of colonies and apoptosis cells were also detected in these groups. PcDNA3.1‐PLK1 cotransfection attenuated the effects of inhibiting colony formation and promoting apoptosis caused by si‐Lnc or miR‐593 mimics (Figure 6B, 6C; *P* < 0.05). In glioma tissues, the relative expression of PLK1 protein was positively correlated with lncRNA ENST00000413528 (*R* ^2^ = 0.4086, *P* < 0.05). Thus, we could infer from our results that PLK1 mRNA lacking 3′UTR reversed the effects of si‐Lnc or miR‐593 mimics in U251 and LN229 cells, suggesting that the three genes may share the same regulatory pathway.

## 4 DISCUSSION

Recent evidence about lncRNAs has indicated that abnormal lncRNA expression may not only affect the regulation of eukaryotic genomes but also promote the growth of malignant cells, leading to tumor progression.^35^, ^36^ Researchers usually use biological informational analysis, expression level regulation, dual luciferase reporter assay, RNA immunoprecipitation assay, and restore assay to conclude a ceRNA function.^37^, ^38^ The current study provides evidence for overexpressed ENST00000413528’s role as a tumor‐promoting gene in glioma tissues and U251 and LN229 cell lines. As shown in Figures 3A and 4A, the binding sites between lncRNA ENST00000413528 and miR‐593‐5p, miR‐593‐5p and PLK1 are the same. Our experimental results also demonstrated that the effects of the downregulation of ENST00000413528 and upregulation of miR‐593‐5p on glioma cells were consistent; there was a decrease in the expression of the PLK1 protein, inhibition of cell proliferation and colony formation, inducement of the G0/G1 arrest, and promotion of the apoptosis of glioma cells. In view of all of the results in our research, ENST00000413528 was identified as a molecular sponge that attenuated the inhibitory regulation of miR‐593‐5p on PLK1 mRNA by competitive binding, resulting in an increased expression of the PLK1 protein and a promotion effect on cell proliferation.

It is not uncommon for lncRNA to play a role through ceRNA pathways in gliomas, either as a tumor‐promoted gene or a tumor‐inhibitory gene. LncRNA CASC2 was reported to be identified as a glioma suppressor gene via miR‐21, while lncRNA XIST is an oncogene that acts as a molecular sponge of miR‐429.^39^, ^40^ Among numerous kinds of nonprotein‐coding RNAs, lncRNAs have a key regulatory role in cancer biology. LncRNAs are dysregulated in different types of cancer and the expression levels of certain lncRNAs are associated with metastasis and prognosis of cancer. MiR‐593 has been reported to be related to cisplatin sensitivity in tongue squamous cell carcinoma and radiosensitization in nasopharyngeal carcinoma cells.^41^, ^42^ The miR‐593 and PLK1 interaction were investigated in esophageal carcinoma,^43^ the tumor‐promoted effect was similar to that seen in our study on glioma cells. Our study links lncRNA ENST00000413528 to miR‐593 and PLK1 and demonstrates their possible interaction mechanism, which may help to better understand the multiple functions of lncRNAs and provide some experimental basis for the possible clinical application in the later stage.

Consistent with our findings, PLK1 was reported to be upregulated in glioma tissues and cells^44^; knockdown of PLK1 inhibits invasion and promotes apoptosis in U251 and U87 glioma cells through regulating autophagy. In addition, PLK1 downregulation has also been reported to enhance the sensitivity of glioma cells to cisplatin or irradiation.^45^, ^46^ Several PLK1 inhibitors are currently in clinical trials as cancer therapeutics.^47^ BI2536, one of the PLK1 inhibitors, was found to enhance the inhibition of temozolomide(TMZ) on glioma stem cells^48^; BI 6727, a new generation, highly selective inhibitor of PLK1, resulted in decreased cell proliferation and a marked increase in Diffuse Intrinsic Pontine Glioma cellular apoptosis.^49^ These findings are absolutely interesting and demonstrate a potential for clinical use of PLK1 targeted therapy. In our study, we designed our research from another aspect. We found a new functional lncRNA ENST00000413528, which may regulate PLK1 expression via miR‐593‐5p. Different with PLK1 inhibitors, our research enriched the upstream regulatory network mechanism of PLK1, and may provide some fundamental evidence for further PLK1 pathway studies.

Taken together, our results reveal a regulatory axis of lncRNA ENST00000413528/miR‐593/PLK1 in glioma, to our knowledge, this is the first research project of lncRNA ENST00000413528 expression profile in glioma. These findings could help us enrich the knowledge on the mechanism of glioma carcinogenesis and find new diagnostic or therapeutic targets related to lncRNA ENST00000413528, miR‐593‐5p, or PLK1 for glioma. However, the roles of lncRNAs in gliomas are diverse and the molecular pathway networks involved are also very complex, further studies are also necessary to elucidate to fully understand the mechanisms.

## 5 CONCLUSION

LncRNA ENST00000413528 is overexpressed in both human glioma tissues and U251 and LN229 cells; additionally, the knockdown of ENST00000413528 in glioma cells inhibits cell proliferation and colony formation abilities and induces the G0/G1 arrest of the cell cycle, and promotes apoptosis via a miR‐593‐5p/PLK1 pathway.

## CONFLICT OF INTEREST

The authors declare no conflict of interest.
